# Prognostic impact of sepsis-induced coagulopathy on mortality and clinical outcomes in ICU patients

**DOI:** 10.1186/s12871-026-04104-3

**Published:** 2026-07-18

**Authors:** Ayşe Çapar, Derya Özyiğitoğlu, Cemre Abacı, Şeyma Başlılar

**Affiliations:** 1 Anaesthesiology and Intensive Care Medicine, Sultan Abdulhamid Han Training and Research Hospital, Istanbul, Turkey; 2 Infectious Diseases and Clinical Microbiology, Sultan Abdulhamid Han Training and Research Hospital, Istanbul, Turkey; 3 Chest Diseases, Sultan Abdulhamid Han Training and Research Hospital, Istanbul, Turkey

**Keywords:** Sepsis, Sepsis-induced coagulopathy, Mortality, Intensive care unit, Organ support, Prognosis

## Abstract

**Objective:**

This study aimed to assess the prognostic impact of sepsis-induced coagulopathy (SIC) on mortality and clinical outcomes in patients with sepsis admitted to the intensive care unit (ICU).

**Materials and methods:**

This retrospective cohort study included 1,305 adult patients with sepsis admitted to a tertiary pulmonary ICU between January 2016 and May 2025. SIC was defined using the International Society on Thrombosis and Haemostasis (ISTH) criteria, based on platelet (PLT) count, prothrombin time/international normalized ratio (PT/INR), and total Sequential Organ Failure Assessment (SOFA) score components. The primary outcome was in-hospital mortality, and secondary outcomes included vasopressor requirement, escalation to second-line vasopressor therapy, invasive mechanical ventilation (IMV), and continuous renal replacement therapy (CRRT). Multivariable logistic regression and receiver operating characteristic (ROC) analyses were conducted to assess the prognostic value of SIC.

**Results:**

The prevalence of SIC was 19.2%. Patients with SIC had significantly higher in-hospital mortality than those without SIC (79.3% vs. 60.6%, *p* < 0.001). Vasopressor use and escalation to second-line vasopressor therapy were more frequent in the SIC-positive group (98.0% vs. 92.5%, *p* = 0.001; 45.8% vs. 31.3%, *p* < 0.001, respectively). In multivariable analyses, both SIC positivity (OR: 2.172, 95% CI: 1.528–3.087, *p* < 0.001) and the SIC score (OR: 1.324, 95% CI: 1.175–1.492, *p* < 0.001) were independently associated with mortality. ROC analysis showed that the SIC score had moderate discriminative performance for predicting mortality (AUC: 0.619, 95% CI: 0.588–0.649, *p* < 0.001), with an SIC score ≥ 4 identified as the clinically interpreted threshold.

**Conclusion:**

SIC was independently associated with higher in-hospital mortality and greater need for vasopressor support in critically ill patients with sepsis. These findings suggest that SIC may provide complementary prognostic information when interpreted alongside established severity assessment tools; however, prospective studies are needed to clarify its clinical relevance and potential role in patient management.

**Supplementary Information:**

The online version contains supplementary material available at https://doi.org/10.1186/s12871-026-04104-3.

## Introduction

Sepsis is a complex and heterogeneous clinical syndrome marked by life-threatening organ dysfunction caused by an abnormal host response to infection [1]. Despite advances in intensive care management, sepsis remains a major global health issue linked to high rates of morbidity and mortality [1, 2]. In sepsis pathogenesis, there is a close, complex interaction between the inflammatory and coagulation pathways. The infection-triggered thromboinflammatory response causes endothelial damage, platelet activation, and reduced fibrinolysis; together, these processes lead to microvascular thrombosis, impairing tissue perfusion and contributing to organ dysfunction [3, 4].

Sepsis-induced coagulopathy (SIC) is considered an early stage of disseminated intravascular coagulation (DIC) and indicates a clinical condition that allows for early identification of high-risk patient groups [4, 5]. DIC is characterized by widespread microvascular thrombosis, consumption of coagulation factors, and the simultaneous occurrence of thrombotic and hemorrhagic complications, and it is associated with high mortality [5]. Therefore, early detection of coagulopathy is essential for evaluating sepsis severity and forecasting prognosis. The SIC scoring system, which relies on routine clinical and laboratory parameters and is simple to use, was created to recognize early-stage DIC and has gained approval in international clinical practice [6, 7].

Recent studies have underscored the importance of accessible, dynamic biomarkers for early risk stratification and prognostic assessment in critically ill patients with sepsis [8]. In this context, coagulation-based prognostic tools have attracted growing attention because activation of coagulation pathways plays a central role in sepsis-associated organ dysfunction and mortality. Although previous studies have demonstrated an association between SIC and adverse outcomes in sepsis [9, 10], important gaps remain in the current literature. Existing studies have primarily focused on mortality outcomes, while the relationship between SIC and organ support requirements in critically ill populations has been less clearly characterized. In addition, data evaluating the prognostic relevance of SIC in real-world cohorts of patients requiring advanced supportive therapies, such as vasopressor escalation, invasive mechanical ventilation (IMV), and continuous renal replacement therapy (CRRT), remain limited.

Therefore, the primary aim of this study was to evaluate the association between SIC and in-hospital mortality among patients with sepsis admitted to the intensive care unit (ICU). The secondary aims were to examine the relationship between SIC and organ support requirements, including vasopressor therapy, escalation to second-line vasopressor treatment, IMV, CRRT, and ICU length of stay (LOS). This study aimed to further clarify the prognostic relevance of SIC in a large real-world ICU cohort and to assess its potential contribution to risk stratification in critically ill patients with sepsis.

## Materials and methods

### Study design and setting

This retrospective observational cohort study aimed to assess the impact of SIC on mortality and clinical outcomes in patients diagnosed with sepsis and admitted to the respiratory ICU at Sultan Abdulhamid Han Training and Research Hospital. Adult patients who were admitted to the respiratory ICU between January 2016 and May 2025 and diagnosed with sepsis according to the Sepsis-3 criteria were included in the study.

### Patient selection

**Fig. 1:**
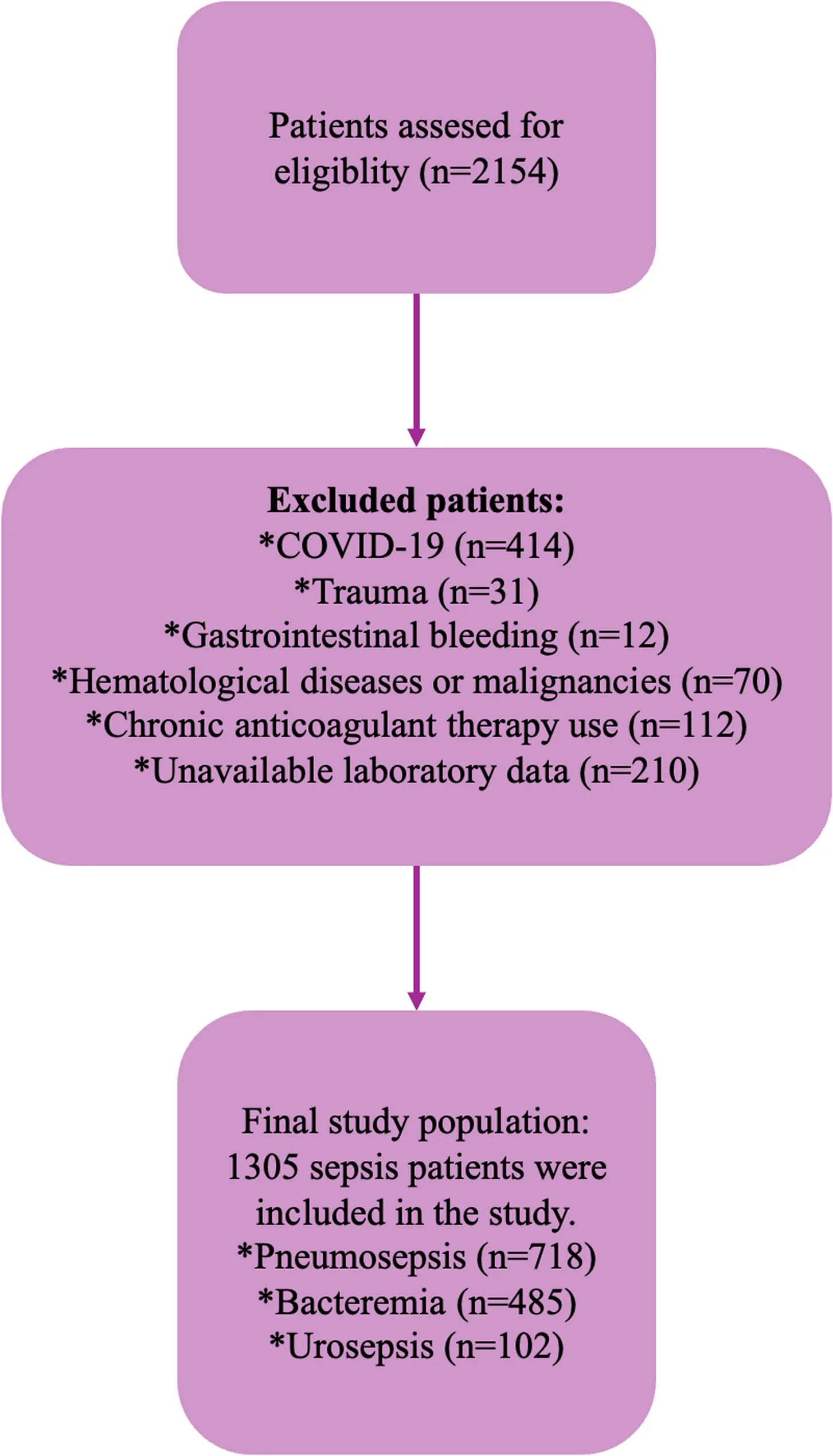
Study Flowchart. COVID-19: Coronavirus Disease 2019

Patients aged 18 years and older who were monitored in the ICU for at least 24 h and had a confirmed diagnosis of sepsis were included in the study. Patients with missing data on coagulation parameters, those with active hematological malignancies, those on chronic anticoagulant therapy, those who experienced major bleeding during their stay in the ICU, and those admitted to the ICU due to trauma were excluded. Patients with coronavirus disease-2019 (COVID-19) were also excluded because COVID-19 is associated with a distinct thromboinflammatory and coagulopathic profile that may independently affect coagulation parameters and clinical outcomes [11]. Repeated admissions of the same patient were not included in the analyses (Fig. 1).

### Data collection

Demographic characteristics, comorbidities, vital signs, and laboratory parameters were retrospectively collected from the electronic medical record system. Laboratory parameters measured within the first 24 h after the diagnosis of sepsis—including hemoglobin (Hgb), white blood cell (WBC) count, and platelet (PLT) count—as well as prothrombin time expressed as the International Normalized Ratio (PT/INR), fibrinogen, D-dimer, C-reactive protein (CRP), procalcitonin (PCT), lactate levels, and other biochemical parameters, were recorded. Disease severity was evaluated using the Acute Physiology and Chronic Health Evaluation (APACHE II) and Sequential Organ Failure Assessment (SOFA) scores. Additionally, the need for vasopressor therapy and IMV within the first 48 h following sepsis diagnosis was identified through review of patient records and documented accordingly. The requirement for a second vasopressor was defined as occurring when the dose of the first vasopressor, noradrenaline, exceeded 0.25 µg/kg/min [12]. CRRT requirement was assessed during the first 7 days of ICU follow-up.

Patients with missing coagulation parameter data were excluded because these variables were required to calculate the SIC score. For the remaining study variables, missing data were handled using complete-case analysis (listwise deletion) in statistical analyses. Accordingly, patients with missing values for variables included in a specific analysis were excluded only from that analysis.

### Definition of SIC

SIC was assessed using the SIC scoring system established by the International Society on Thrombosis and Haemostasis (ISTH) [4]. SIC positivity was defined as meeting two primary criteria. Specifically, the total SIC score, calculated from PLT, PT/INR, and the total SOFA score, had to be at least 4. Additionally, the subscore based on PT/INR and PLT parameters needed to be at least 3 points. The total SOFA score was determined based on four components: renal, hepatic, respiratory, and cardiovascular systems [13] (Table 1).

**Table 1:** The SIC scoring chart

Category	Parameter	0 point	1 point	2 point
Prothrombin time / International Normalized Ratio	PT/INR	≤1.2	> 1.2–≥1.4	> 1.4
Coagulation	Platelet count (⋅10^9^/L)	≥150	≥100 - <150	< 100
Total SOFA score	SOFA with 4 items	0	1	≥2

The total SOFA score is calculated as the sum of the respiratory, cardiovascular, hepatic, and renal component scores

*SIC* Sepsis-Induced Coagulopathy, *SOFA* Sequential Organ Failure Assessment

### Outcomes

The primary outcome of the study was in-hospital mortality. Secondary outcomes reflected the clinical course and included organ support needs. Specifically, the study evaluated the need for vasopressor therapy within the first 48 h, escalation of vasopressor treatment (defined as requiring a second vasopressor with a dose exceeding the first, noradrenaline to 0.25 µg/kg/min), the need for IMV within 48 h, and the requirement for CRRT during the first 7 days. Additionally, the ICU LOS was analyzed.

### Statistical analysis

Statistical analyses were conducted using Statistical Package for the Social Sciences (SPSS) software (version 30). The distribution of continuous variables was evaluated through both visual methods (histograms and probability plots) and statistical tests (Kolmogorov–Smirnov or Shapiro–Wilk tests). Variables with a normal distribution were reported as mean ± standard deviation, while non-normally distributed variables were presented as median (Q1–Q3). Categorical variables were summarized with counts and percentages.

For group comparisons, the Student’s t-test or Mann–Whitney U test was used for continuous data, and the chi-square test or Fisher’s exact test was applied for categorical data. Multivariable logistic regression analysis was performed to identify independent predictors of in-hospital mortality, with three different models constructed. Model fit was assessed using the Hosmer–Lemeshow goodness-of-fit test.

To assess the incremental prognostic value of the SIC score beyond APACHE II, the predictive performance of APACHE II alone, the SIC score alone, and the combined APACHE II + SIC model was evaluated using receiver operating characteristic (ROC) curve analysis. Discriminative performance was compared using the area under the ROC curve (AUC), and correlated ROC curves were compared using the DeLong test. Model calibration was assessed with the Hosmer–Lemeshow goodness-of-fit test, and explained variance was evaluated using the Nagelkerke R² statistic. The optimal cutoff for the SIC score was determined using the Youden index. A two-sided p-value < 0.05 was considered statistically significant.

### Ethical approval

The study protocol received approval from the Non-Interventional Clinical Research Ethics Committee at Istanbul Sureyyapasa Chest Diseases and Chest Surgery Training and Research Hospital (date:15.05.2025, protocol code: 2024-55). Because of the retrospective design, informed consent was waived. The study was carried out following the principles outlined in the Declaration of Helsinki.

## Results

### Patient characteristics and overall findings

A total of 1,305 patients with sepsis were included in the study. The median age was 68.00 years (56.25–77.75), and 55.8% of patients were male. The in-hospital mortality rate was 64.2%. The need for vasopressor therapy within the first 48 h was observed in 93.6% of patients, the need for IMV in 90%, and the need for CRRT within the first 7 days in 39.9%. The median APACHE II score was 28.00 (23.00–34.00), and the SOFA score was 10.00 (7.00–13.00). The prevalence of SIC was 19.2%. The median ICU LOS was 6.67 (2.89–13.31) days. (Table 2).

**Table 2:** Baseline clinical characteristics, laboratory findings, and clinical outcomes of the study population

Variables	Mean±SD, Median (Q1-Q3) or *n* (%)
Gender	
Male	728 (55.8)
Female	577 (44.2)
Age	68.00 (56.25–77.75)
Presence of ≥1 comorbidity	297 (22.8)
pH	7.31±0.14
BE mmol/L	-6.01±7.95
HCO_3_^−^ mmol/L	19.86±6.01
PO_2_ mmHg	102.50 (68.73–146.00)
PCO_2_ mmHg	39.40 (32.13–46.83)
Lactate mmol/L	1.90 (1.30–3.58)
WBC ⋅10^9^/L	12.16 (7.99–18.96)
Hgb g/dl	10.60 (8.25–11.88)
PLT ⋅10^9^/L	248.50 (153.00-328.75)
Lymphocyte ⋅10^9^/L	0.78 (0.42–1.18)
Neutrophil ⋅10^9^/L	10.20 (6.79–17.28)
Na^+^ mmol/L	137.00 (133.00-141.00)
K^+^ mmol/L	4.11 (3.55–4.80)
Ca^2+^ mg/dl	8.21 (7.75–8.80)
Urea mg/dl	72.40 (46.75-122.98)
Creatinine mg/dl	1.61 (0.91–2.69)
Total bilirubin mg/dl	0.61 (0.37–1.38)
CRP mg/dl	123.50 (50.75-238.77)
PCT ng/ml	1.80 (0.64–17.48)
Albumin g/L	29.33±5.95
Pro-BNP pg/ml	5494.50 (1451.50-18067.25)
AST U/L	34.00 (19.03–63.88)
ALT U/L	20.70 (10.75–50.78)
LDH U/L	269.00 (214.25–427.50)
D-dimer mg/L	2.82 (1.52–6.32)
PT/INR	1.33 (1.21–1.56)
Fibrinogen mg/dl	501.78 (368.25-677.25)
MAP mmHg	72.00 (64.00-79.75)
SpO_2_	96.00 (91.00–99.00)
HR	103.17 (87.08-120.33)
APACHE II	28.00 (23.00–34.00)
SOFA	10.00 (7.00–13.00)
SOFA (with 4 items)	10.00 (6.00–13.00)
PLT + PT/INR subscore	2.00 (1.00–2.00)
SIC score	4.00 (3.00–4.00)
SIC (+)	251 (19.2)
In-hospital mortality	838 (64.2)
IMV need within first 48 h	1174 (90)
CRRT need within first 7 days	521 (39.9)
VP need within first 48 h	1221 (93.6)
Requirement for a second VP	445 (34.1)
ICU LOS days	6.67 (2.89–13.31)

*BE* Base excess, *HCO3-* Bicarbonate, *PaO2* Partial pressure of oxygen, *PaCO2* Partial pressure of carbon dioxide, *Na+* Sodium, *K+* Potassium, *Ca*^2^*+* Calcium, *CRP* C-reactive protein, *PCT* Procalcitonin, *WBC* White blood cell count, *Hgb* Hemoglobin, *PLT* Platelet count, *Pro-BNP* Pro-B-type natriuretic peptide, *AST* Aspartate aminotransferase, *ALT* Alanine aminotransferase, *LDH* Lactate dehydrogenase, *PT/INR* Prothrombin time/International normalized ratio, *MAP* Mean arterial pressure, *SpO2* Peripheral oxygen saturation, *HR* Heart rate, *APACHE II* Acute physiology and chronic health evaluation II, *SOFA* Sequential organ failure assessment, *SIC* Sepsis-induced coagulopathy, *IMV* Invasive mechanical ventilation, *CRRT* Continuous renal replacement therapy, *VP* Vasopressor, *ICU LOS* Length of stay in the intensive care unit

### Clinical and laboratory findings based on SIC status

In-hospital mortality was significantly higher in patients who developed SIC (79.3% vs. 60.6%, *p* < 0.001). The requirement for vasopressors within the first 48 h (98.0% vs. 92.5%, *p* = 0.001) and the need for a second vasopressor (45.8% vs. 31.3%, *p* < 0.001) were also significantly higher in the SIC (+) group. Although the need for IMV did not differ statistically between the groups (86.9% vs. 90.7%, *p* = 0.068). Similarly, the requirement for CRRT was higher in the SIC (+) group; however, this difference was not statistically significant (43.4% vs. 39.1%, *p* = 0.207).

In terms of coagulation parameters, patients with SIC showed significant thrombocytopenia (44.50 ⋅10^9^/L [35.00–88.75] vs. 271.50 ⋅10^9^/L [191.00–347.75], *p* < 0.001) and higher PT/INR (1.59 [1.37–2.00] vs. 1.32 [1.20–1.49], *p* < 0.001) values. Additionally, the APACHE II (30.78 ± 10.26 vs. 27.34 ± 8.47, *p* < 0.001) and SOFA scores (13.50 [10.50–16.25] vs. 10.00 [6.00–13.00], *p* < 0.001) were significantly higher in the SIC (+) group. Other laboratory and clinical findings are detailed in Table 3.

**Table 3:** Clinical and laboratory characteristics of patients according to SIC status

Variables	SIC (+) Mean±SD, Median (Q1-Q3) or *n* (%) *n* = 251	SIC (-) Mean±SD, Median (Q1-Q3) or *n* (%) *n* = 1054	*p*-value
Gender			
Male	145 (57.8)	583 (55.3)	
Female	106 (42.2)	471 (44.7)	0.481
Age	61.50 (54.00-76.25)	69.50 (57.75-78.00)	0.137
Presence of ≥1 comorbidity	52 (20.7)	245 (23.2)	0.391
pH	7.35±0.15	7.30±0.14	0.455
BE mmol/L	-5.86±8.17	-6.05±7.96	**< 0.001**
HCO_3_^−^ mmol/L	20.81±5.87	19.67±6.05	**< 0.001**
PO_2_ mmHg	105.00 (68.00-146.25)	102.20 (68.03–146.50)	**0.001**
PCO_2_ mmHg	37.95 (28.93–42.25)	39.50 (32.18–47.15)	**< 0.001**
Lactate mmol/L	2.65 (1.58–6.28)	1.80 (1.20–3.43)	**< 0.001**
WBC ⋅10^9^/L	8.20 (4.61–18.19)	12.42 (8.39–19.83)	**< 0.001**
Hgb g/dl	10.65 (8.43–13.23)	10.60 (8.10-11.83)	**< 0.001**
PLT ⋅10^9^/L	44.50 (35.00-88.75)	271.50 (191.00-347.75)	**< 0.001**
Lymphocyte ⋅10^9^/L	0.56 (0.30–0.89)	0.87 (0.45–1.21)	**< 0.001**
Neutrophil ⋅10^9^/L	7.93 (3.49–16.50)	10.59 (7.03–17.72)	**< 0.001**
Na^+^ mmol/L	138.00 (131.75-141.25)	137.00 (133.00-141.00)	0.525
K^+^ mmol/L	3.97 (3.27–4.30)	4.23 (3.61–5.01)	**0.022**
Ca^2+^ mg/dl	8.00 (7.44–8.19)	8.30 (7.79–8.90)	**< 0.001**
Urea mg/dl	82.25 (51.75-139.83)	72.00 (41.88-121.65)	**< 0.001**
Creatinine mg/dl	1.90 (1.49–3.30)	1.50 (0.91–2.61)	**0.009**
Total bilirubin mg/dl	1.03 (0.51–5.10)	0.58 (0.35–1.14)	**< 0.001**
CRP mg/dl	175.55 (116.75-324.53)	117.25 (40.20-221.75)	**0.031**
PCT ng/ml	29.40 (5.98–87.29)	1.44 (0.46–11.03)	**< 0.001**
Albumin g/L	26.31±5.42	29.94±5.89	**< 0.001**
Pro-BNP pg/ml	12788.00 (5481.50-35000.00)	4440.00 (1385.00-15341.25)	**< 0.001**
AST U/L	48.05 (31.90-412.70)	28.40 (17.50-62.33)	**< 0.001**
ALT U/L	28.85 (17.70-174.75)	19.30 (10.00-49.20)	**< 0.001**
LDH U/L	409.50 (279.25–656.50)	259.00 (202.50–371.00)	**< 0.001**
D-dimer mg/L	3.46 (1.60–7.36)	2.74 (1.45–5.91)	**< 0.001**
PT/INR	1.59 (1.37-2.00)	1.32 (1.20–1.49)	**< 0.001**
Fibrinogen mg/dl	518.00 (407.25–705.00)	486.50 (364.75-671.75)	**< 0.001**
MAP mmHg	73.50 (57.75–81.50)	72.00 (64.75–79.25)	**0.029**
SpO_2_	95.70 (90.78–98.48)	96.00 (91.00–99.00)	0.932
HR	114.57±27.68	101.53±21.79	0.382
APACHE II	30.78±10.26	27.34±8.47	**< 0.001**
SOFA	13.50 (10.50-16.25)	10.00 (6.00–13.00)	**< 0.001**
SOFA with 4 items	12.00 (7.50–13.50)	10.00 (6.00–13.00)	**< 0.001**
PLT + PT/INR subscore	3.00 (3.00–4.00)	1.00 (0.00–2.00)	**< 0.001**
SIC score	5.00 (5.00–6.00)	3.00 (2.00–4.00)	**< 0.001**
In-hospital mortality	199 (79.3)	639 (60.6)	**< 0.001**
IMV need within first 48 h	218 (86.9)	956 (90.7)	0.068
CRRT need within first 7 days	109 (43.4)	412 (39.1)	0.207
VP need within first 48 h	246 (98.0)	975 (92.5)	**0.001**
Requirement for a second VP	115 (45.8)	330 (31.3)	**< 0.001**
ICU LOS days	6.17 (1.82–15.60)	6.79 (3.27–13.19)	**< 0.001**

*BE* Base excess, *HCO3-* Bicarbonate, *PaO2* Partial pressure of oxygen, *PaCO2* Partial pressure of carbon dioxide, *Na+* Sodium, *K+* Potassium, *Ca*^2^*+* Calcium, *CRP* C-reactive protein, *PCT* Procalcitonin, *WBC* White blood cell count, *Hgb* Hemoglobin, *PLT* Platelet count, *Pro-BNP* Pro-B-type natriuretic peptide, *AST* Aspartate aminotransferase, *ALT* Alanine aminotransferase, *LDH* Lactate dehydrogenase, *PT/INR* Prothrombin time/International normalized ratio, *MAP* Mean arterial pressure, *SpO2* Peripheral oxygen saturation, *HR* Heart rate, *APACHE II* Acute Physiology and Chronic Health Evaluation II, *SOFA* Sequential Organ Failure Assessment. SIC: Sepsis-Induced Coagulopathy, *IMV* Invasive mechanical ventilation, *CRRT* Continuous renal replacement therapy, *VP* Vasopressor, *ICU LOS* Length of stay in the intensive care unit

### Clinical and laboratory findings related to mortality development

Patients who experienced in-hospital mortality were older (70.00 years [58.50−77.00] vs. 66.00 years [54.00−78.00], *p* < 0.001) and had lower PLT counts (241.00 ⋅10^9^/L [122.00–302.00] vs. 269.00 ⋅10^9^/L [162.00–347.00], *p* < 0.001). PT/INR levels (1.39 [1.24–1.84] vs. 1.31 [1.17–1.48], *p* < 0.001) were also significantly higher in these patients. The need for CRRT (47.0% vs. 27.2%, *p* < 0.001), initial vasopressor use (99.0% vs. 83.7%, *p* < 0.001), and second vasopressor use (48.9% vs. 7.5%, *p* < 0.001) were also significantly greater among patients who died in the hospital.

Additionally, APACHE II score (33.00 [27.00–36.00] vs. 24.00 [18.00–29.00], *p* < 0.001) and SOFA score (13.00 [10.00–14.50] vs. 8.00 [4.00–10.00], *p* < 0.001 were significantly higher among patients who died. SIC positivity was also significantly higher among patients who died (23.7% vs. 11.1%, *p* < 0.001). Other laboratory and clinical findings are detailed in Table 4.

**Table 4:** Clinical and laboratory characteristics according to in-hospital mortality

Variables	Non-survivors Mean±SD, Median (Q1-Q3) or *n* (%) *n* = 838	Survivors Mean±SD, Median (Q1-Q3) or *n* (%) *n* = 467	*p*-value
Gender			
Male	446 (53.2)	282 (60.4)	
Female	392 (46.8)	185 (39.6)	**0.012**
Age	70.00 (58.50–77.00)	66.00 (54.00–78.00)	**< 0.001**
Presence of ≥1 comorbidity	207 (24.7)	90 (19.3)	**0.025**
pH	7.32±0.14	7.29±0.14	**< 0.001**
BE mmol/L	-5.41±7.78	-6.60±8.14	**< 0.001**
HCO_3_^−^ mmol/L	20.30±6.10	19.44±5.95	**< 0.001**
PO_2_ mmHg	97.40 (58.25–144.50)	110.00 (70.30–148.00)	0.231
PCO_2_ mmHg	39.40 (32.15–47.10)	39.30 (31.00-46.90)	0.262
Lactate mmol/L	2.30 (1.30–4.65)	1.70 (1.30–3.10)	**< 0.001**
WBC ⋅10^9^/L	11.36 (7.81–18.63)	12.50 (8.38–20.09)	0.293
Hgb g/dl	9.60 (8.00-11.20)	11.20 (9.40–12.50)	**< 0.001**
PLT ⋅10^9^/L	241.00 (122.00-302.00)	269.00 (162.00-347.00)	**< 0.001**
Lymphocyte ⋅10^9^/L	0.84 (0.46–1.29)	0.70 (0.38–1.08)	0.765
Neutrophil ⋅10^9^/L	8.88 (6.65–15.84)	10.94 (7.27–17.70)	0.446
Na^+^ mmol/L	138.00 (131.00-141.00)	137.00 (133.00-140.00)	0.198
K^+^ mmol/L	4.04 (3.53–4.73)	4.14 (3.70–4.83)	0.017
Ca^2+^ mg/dl	8.10 (7.44–8.77)	8.26 (7.94–8.86)	0.238
Urea mg/dl	87.10 (52.55–128.00)	68.20 (33.80-117.40)	**< 0.001**
Creatinine mg/dl	1.78 (0.96–2.82)	1.30 (0.82–2.69)	**< 0.001**
Total bilirubin mg/dl	0.71 (0.49–2.34)	0.56 (0.34–0.92)	**< 0.001**
CRP mg/dl	129.00 (76.50-245.50)	97.00 (26.38–217.50)	0.311
PCT ng/ml	2.84 (1.05–22.51)	1.20 (0.37–13.10)	0.218
Albumin g/L	28.24±6.08	30.38±5.68	**< 0.001**
Pro-BNP pg/ml	7020.00 (2998.00-24704.00)	4038.00 (1203.00-15300.00)	**< 0.001**
AST U/L	35.00 (23.50-98.75)	24.50 (16.80–53.30)	**0.001**
ALT U/L	23.00 (14.90-50.05)	18.00 (10.00-52.40)	0.561
LDH U/L	289.00 (236.00-551.00)	255.00 (201.00-323.00)	**< 0.001**
D-dimer mg/L	3.22 (1.59–6.51)	2.40 (1.12–5.10)	**0.004**
PT/INR	1.39 (1.24–1.84)	1.31 (1.17–1.48)	**< 0.001**
Fibrinogen mg/dl	477.00 (353.00-604.50)	531.00 (384.00-691.00)	**0.002**
MAP mmHg	73.00 (62.00–79.00)	72.00 (64.00–80.00)	**0.025**
SpO_2_	95.00 (90.00–99.00)	96.50 (93.00–99.00)	**< 0.001**
HR	105.16±25.73	102.30±20.72	0.358
APACHE II	33.00 (27.00–36.00)	24.00 (18.00–29.00)	**< 0.001**
SOFA	13.00 (10.00-14.50)	8.00 (4.00–10.00)	**< 0.001**
SOFA with 4 items	13.00 (10.00–14.00)	7.00 (4.00–10.00)	**< 0.001**
PLT + PT/INR subscore	2.00 (1.00–2.00)	1.00 (0.00–2.00)	**< 0.001**
SIC score	4.00 (3.00–4.00)	3.00 (2.00–4.00)	**< 0.001**
SIC (+)	199 (23.7)	52 (11.1)	**< 0.001**
IMV need within first 48 h	738 (88.1)	436 (93.4)	**0.002**
CRRT need within first 7 days	394 (47.0)	127 (27.2)	**< 0.001**
VP need within first 48 h	830 (99.0)	391 (83.7)	**< 0.001**
Requirement for a second VP	410 (48.9)	35 (7.5)	**< 0.001**
ICU LOS days	6.08 (2.73–16.17)	6.88 (2.92–9.17)	**< 0.001**

*BE* Base excess, *HCO3-*: Bicarbonate, *PaO2* Partial pressure of oxygen, *PaCO2* Partial pressure of carbon dioxide, *Na+* Sodium, *K+* Potassium, *Ca*^2^*+* Calcium, *CRP* C-reactive protein, *PCT* Procalcitonin, *WBC* White blood cell count, *Hgb* Hemoglobin, *PLT* Platelet count, *Pro-BNP* Pro-B-type natriuretic peptide, *AST* Aspartate aminotransferase, *ALT* Alanine aminotransferase, *LDH* Lactate dehydrogenase, *PT/INR* Prothrombin time/International normalized ratio, *MAP* Mean arterial pressure, *SpO2* Peripheral oxygen saturation, *HR* Heart rate, *APACHE II* Acute physiology and chronic health evaluation II, *SOFA* Sequential organ failure assessment, *SIC* Sepsis-induced coagulopathy, *IMV* Invasive mechanical ventilation, *CRRT* Continuous renal replacement therapy, *VP* Vasopressor, *ICU LOS* Length of stay in the intensive care unit

### Univariate logistic regression analysis

In the univariate analysis, advanced age (OR: 1.023, 95% CI: 1.016−1.030, *p* < 0.001), the need for CRRT (OR: 2.376, 95% CI: 1.860−3.035, *p* < 0.001), initial vasopressor need (OR: 20.166, 95% CI: 9.637−42.198, *p* < 0.001) and second vasopressor therapy (OR: 11.824, 95% CI: 8.166−17.120, *p* < 0.001), elevated lactate levels (OR: 1.186, 95% CI: 1.126−1.249, *p* < 0.001), higher PT/INR (OR: 2.641, 95% CI: 1.966−3.546, *p* < 0.001) and D-dimer levels (OR: 1.038, 95% CI: 1.008−1.070, *p* = 0.014), lower albumin (OR: 0.963, 95% CI: 0.946−0.981, < 0.001), PLT (OR: 0.998, 95% CI: 0.997−0.999, *p* < 0.001), and fibrinogen levels (OR: 0.999, 95% CI: 0.998−1.000, *p* = 0.004), as well as increased APACHE II (OR: 1.133, 95% CI: 1.114−1.151, < 0.001), SOFA (OR: 1.359, 95% CI: 1.310−1.411, < 0.001), and SIC scores (OR: 1.468, 95% CI: 1.325−1.627, < 0.001) were all significantly associated with mortality. SIC positivity was associated with increased odds of in-hospital mortality (OR: 2.485, 95% CI: 1.788–3.455, *p* < 0.001) (Table 5).

**Table 5:** Univariate logistic regression analysis for predictors of in-hospital mortality

Variables	OR (95% CI)	B coefficient	*p*-value
Age	1.023 (1.016–1.030)	0.023	**< 0.001**
Gender	0.746 (0.593–0.939)	-0.292	**0.013**
Lactate mmol/L	1.186 (1.126–1.249)	0.171	**< 0.001**
Hgb g/dl	0.865 (0.822–0.911)	-0.145	**< 0.001**
PLT ⋅ 10^9^/L	0.998 (0.997–0.999)	-0.002	**< 0.001**
Urea mg/dl	1.007 (1.005–1.008)	0.006	**< 0.001**
Creatinine mg/dl	1.125 (1.055-1.200)	0.118	**< 0.001**
Total bilirubin mg/dl	1.111 (1.046–1.180)	0.105	**< 0.001**
Albumin g/L	0.963 (0.946–0.981)	-0.038	**< 0.001**
Pro-BNP pg/ml	1.000 (1.000–1.000)	0.000	**< 0.001**
D-dimer mg/L	1.038 (1.008–1.070)	0.038	**0.014**
PT/INR	2.641 (1.966–3.546)	0.971	**< 0.001**
Fibrinogen mg/dl	0.999 (0.998-1.000)	-0.001	**0.004**
MAP mmHg	1.008 (1.001–1.016)	0.008	**0.033**
SpO2	0.961 (0.942–0.981)	-0.039	**< 0.001**
APACHE II	1.133 (1.114–1.151)	0.125	**< 0.001**
SOFA	1.359 (1.310–1.411)	0.307	**< 0.001**
SOFA with 4 items	1.349 (1.300–1.400)	0.299	**< 0.001**
SIC score	1.468 (1.325–1.627)	0.384	**< 0.001**
SIC (+)	2.485 (1.788–3.455)	0.910	**< 0.001**
CRRT need within first 7 days	2.376 (1.860–3.035)	0.865	**< 0.001**
VP need within first 48 h	20.166 (9.637–42.198)	3.004	**< 0.001**
Requirement for a second VP	11.824 (8.166–17.120)	2.470	**< 0.001**

*Hgb* Hemoglobin, *PLT* Platelet count, *Pro-BNP* Pro-B-type natriuretic peptide, *PT/INR* Prothrombin time/International normalized ratio, *MAP* Mean arterial pressure, *SpO2* Peripheral oxygen saturation, *APACHE II* Acute Physiology and Chronic Health Evaluation II, *SOFA* Sequential Organ Failure Assessment, *SIC* Sepsis-Induced Coagulopathy, *CRRT* Continuous renal replacement therapy, *VP* Vasopressor

### Multivariate logistic regression analysis

In the multivariate analyses conducted across three different models, SIC positivity was independently associated with mortality in Model 1 (OR: 2.172, 95% CI: 1.528–3.087, *p* < 0.001), along with advanced age (OR: 1.024, 95% CI: 1.016−1.031, *p* < 0.001), elevated lactate levels (OR: 1.150, 95% CI: 1.090−1.212, *p* < 0.001), and lower albumin levels (OR: 0.971, 95% CI: 0.953−0.990, *p* = 0.003).

In Model 2, among coagulation parameters, PT/INR (OR: 2.421, 95% CI: 1.584−3.700, < 0.001) and PLT levels (OR: 0.998, 95% CI: 0.997−0.999, *p* = 0.006) were independently associated with mortality, while fibrinogen (OR: 0.999, 95% CI: 0.999−1.000, *p* = 0.221) and D-dimer levels (OR: 1.016, 95% CI: 0.981−1.052, *p* = 0.370) were not significant.

In Model 3, after adjusting for disease severity, advanced age (OR: 1.012, 95% CI: 1.004−1.020, *p* = 0.003), the APACHE II score (OR: 1.120, 95% CI: 1.102−1.139, *p* < 0.001), lactate level (OR: 1.094, 95% CI: 1.034−1.158, *p* = 0.002), and SIC score (OR: 1.324, 95% CI: 1.175−1.492, *p* < 0.001) were independently associated with mortality (Table 6).

**Table 6:** Multivariable logistic regression models for predictors of in-hospital mortality

Variables	Model 1			Model 2			Model 3		
	OR (95% CI)	B coefficient	*p*- value	OR (95% CI)	B coefficient	*p*-value	OR (95% CI)	B coefficient	*p*- value
Age	1.024 (1.016–1.031)	0.023	**< 0.001**	1.020 (1.010–1.031)	0.020	**< 0.001**	1.012 (1.004–1.020)	0.012	**0.003**
PLT				0.998 (0.997–0.999)	-0.002	**0.006**			
PT/INR				2.421 (1.584-3.700)	0.884	**< 0.001**			
Lactate	1.150 (1.090–1.212)	0.139	**< 0.001**				1.094 (1.034–1.158)	0.090	**0.002**
Albumin	0.971 (0.953–0.990)	-0.029	**0.003**						
Fibrinogen				0.999 (0.999-1.000)	-0.001	0.221			
D-dimer				1.016 (0.981–1.052)	0.016	0.370			
APACHE II							1.120 (1.102–1.139)	0.114	**< 0.001**
SIC Score							1.324 (1.175–1.492)	0.281	**< 0.001**
SIC (+)	2.172 (1.528–3.087)	0.776	**< 0.001**						

Model 1: SIC model including age, lactate, albumin, and SIC positivity, Model 2: Coagulation parameters model including age, PLT, PT/INR, fibrinogen, and D-dimer, Model 3: Severity-adjusted model including age, APACHE II score, lactate, and SIC score. VIF < 5, Tolerance > 0.8, Hosmer-Lemeshow test > 0.05 for both

*PLT* Platelet count, *PT/INR* Prothrombin time/International normalized ratio, *APACHE II* Acute Physiology and Chronic Health Evaluation II, *SIC* Sepsis-Induced Coagulopathy

### Incremental prognostic performance of the SIC score

**Fig. 2:**
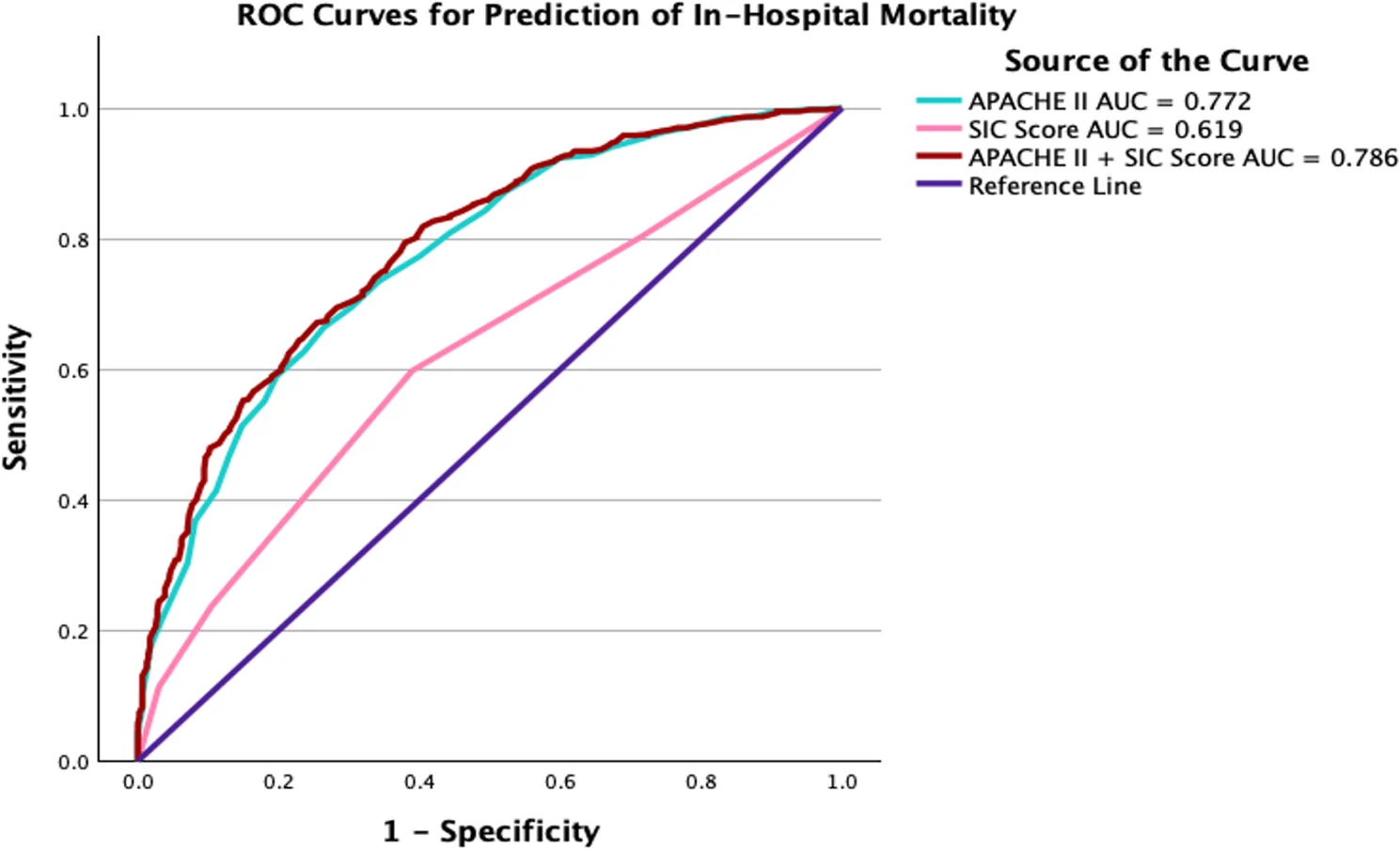
ROC curves comparing APACHE II alone, SIC score alone, and the combined APACHE II + SIC model for predicting in-hospital mortality. The addition of the SIC score to APACHE II resulted in a modest improvement in discriminative performance (AUC: 0.786 vs. 0.772; ΔAUC = 0.014; DeLong test, *p* = 0.002). ROC: Receiver operating characteristic; APACHE II: Acute Physiology and Chronic Health Evaluation; SIC: Sepsis-Induced Coagulopathy

The predictive performance of APACHE II alone, the SIC score alone, and the combined APACHE II + SIC model for in-hospital mortality was compared (Table 7; Fig. 2). The AUCs were 0.772 (95% CI: 0.745–0.798) for APACHE II, 0.619 (95% CI: 0.588–0.649) for the SIC score, and 0.786 (95% CI: 0.761–0.812) for the combined model. The combined model had a higher Nagelkerke R² than APACHE II alone (0.308 vs. 0.282), while the Hosmer–Lemeshow goodness-of-fit p-values were 0.801 and 0.507, respectively. Comparison of the correlated ROC curves showed a statistically significant difference between APACHE II alone and the combined model (DeLong test, *p* = 0.002).

**Table 7:** Comparison of the predictive performance of APACHE II alone, the SIC score alone, and the combined APACHE II + SIC model for predicting in-hospital mortality

Variables	AUC (95% CI)	Nagelkerke *R*^2^	Hosmer-Lemeshow *p*-value	Omnibus *p*-value	*p*-value
APACHE II	0.772 (0.745–0.798)	0.282	0.507	< 0.001	< 0.001
SIC Score	0.619 (0.588–0.649)	0.059	0.011	< 0.001	< 0.001
APACHE + SIC Score	0.786 (0.761–0.812)	0.308	0.801	< 0.001	< 0.001

The addition of the SIC score to APACHE II resulted in a modest but statistically significant improvement in discriminative performance (DeLong test, *p* = 0.002). No evidence of problematic multicollinearity was observed between APACHE II and the SIC score in the combined model (all VIF values < 2.0 and tolerance values > 0.8)

*APACHE II* Acute Physiology and Chronic Health Evaluation II, *SIC* Sepsis-Induced Coagulopathy

No evidence of problematic multicollinearity was observed between APACHE II and the SIC score in the combined model (all VIF values < 2.0 and tolerance values > 0.8).

### Diagnostic performance of the SIC score

In the ROC analysis, the SIC score yielded an AUC of 0.619 (95% CI: 0.588–0.649, *p* < 0.001) for predicting in-hospital mortality. A cut-off value of ≥ 4 yielded a sensitivity of 60% and a specificity of 61% (Table 8; Fig. 2). Because the SIC score is an integer-based system, the statistically derived threshold was clinically interpreted as ≥ 4.

**Table 8:** ROC Analysis of SIC Score for Mortality

Variables	AUC (95% CI)	Cut off value	Sensitivity	Specificity	Youden Index	*p*-value
SIC Score	0.619 (0.588–0.649)	≥4	0.60	0.61	0.208	< 0.001

The Youden index identified a statistical threshold between 3 and 4, which was clinically interpreted as an SIC score ≥ 4 because the SIC score can only take integer values

*SIC* Sepsis-Induced Coagulopathy

### Distribution of survivors and non-survivors according to coagulation parameters and SIC score

**Fig. 3:**
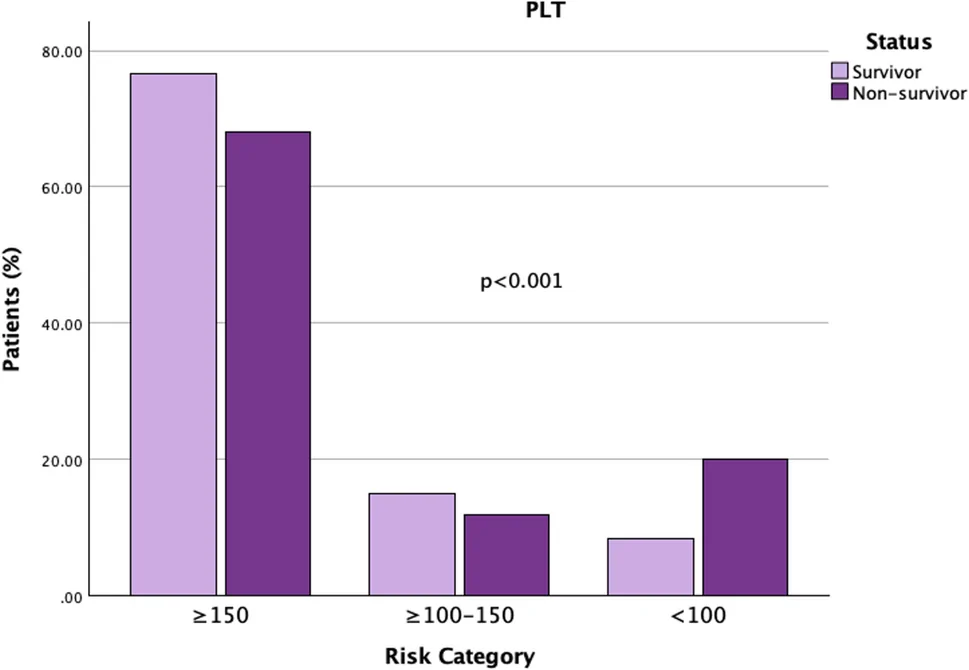
Distribution of patients across predefined PLT risk categories among survivors and non-survivors. Bars show the percentage of patients in each outcome group within each PLT category. The distribution of PLT categories differed significantly between survivors and non-survivors (*p* < 0.001). PLT: Platelet count

**Fig. 4:**
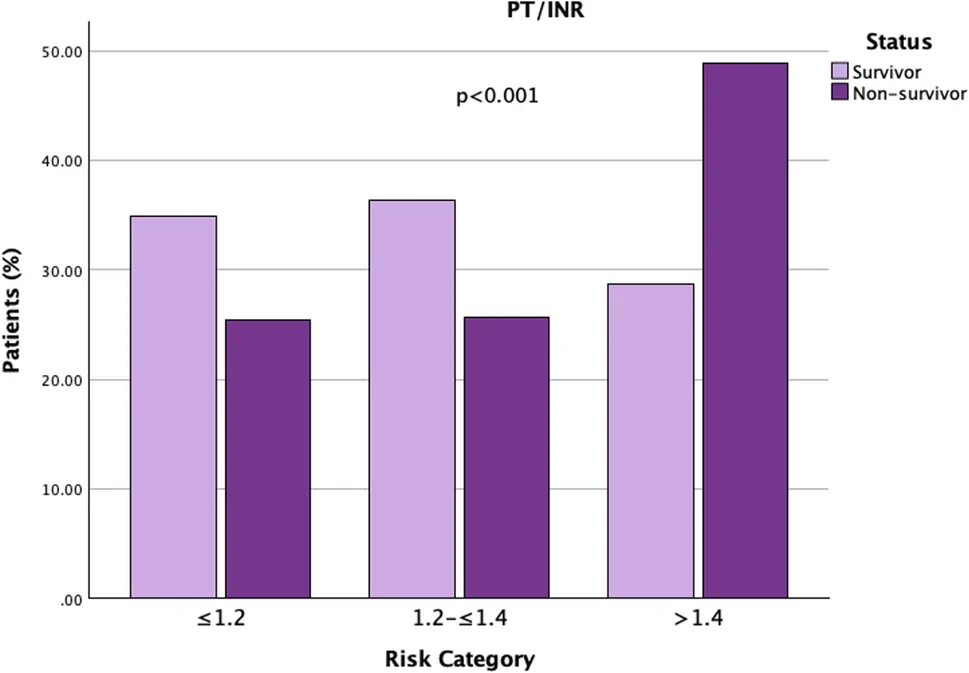
Distribution of patients across predefined PT/INR risk categories among survivors and non-survivors. Bars show the percentage of patients in each outcome group within each PT/INR category. The distribution of PT/INR categories differed significantly between survivors and non-survivors (*p* < 0.001). PT/INR: Prothrombin Time/International Normalized Ratio

**Fig. 5:**
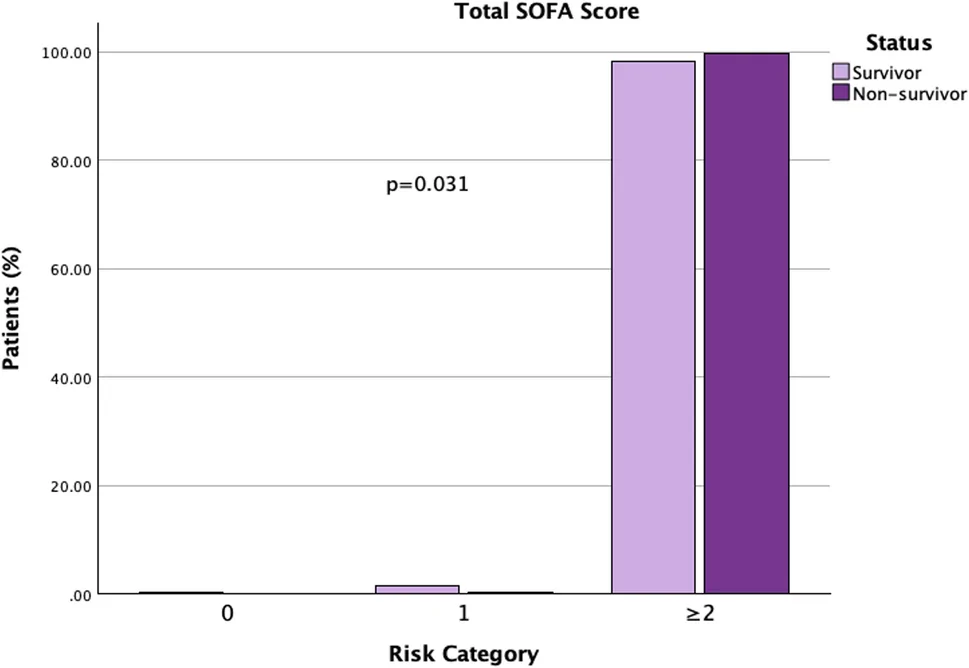
Distribution of patients across predefined total SOFA score categories among survivors and non-survivors. Bars show the percentage of patients in each outcome group within each SOFA score category. The distribution of SOFA score categories differed significantly between survivors and non-survivors (*p* = 0.031). SOFA: Sequential Organ Failure Assessment

**Fig. 6:**
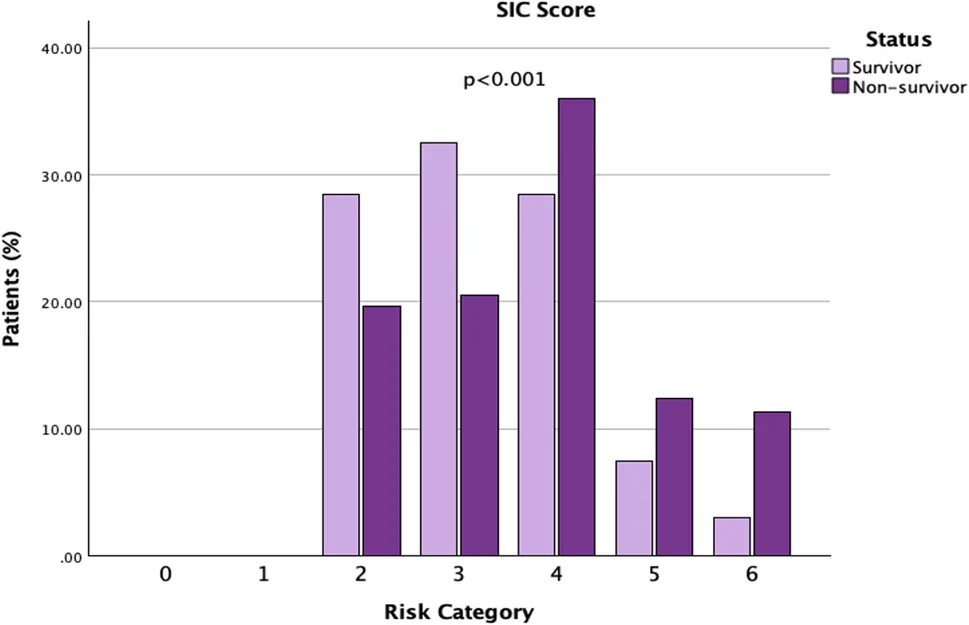
Distribution of patients by SIC score category among survivors and non-survivors. Bars show the percentage of patients in each outcome group within each SIC score category. The distribution of SIC score categories differed significantly between survivors and non-survivors (*p* < 0.001). SIC: Sepsis-Induced Coagulopathy

The distributions of survivors and non-survivors across predefined coagulation parameters and disease severity score categories are shown in Figs. 3, 4, 5 and 6. Significant differences in the distributions of PLT, PT/INR, total SOFA, and SIC score categories were observed between survivors and non-survivors (all *p* < 0.05).

### Exploratory analysis of COVID-19-associated sepsis and sepsis-induced coagulopathy

In an additional exploratory analysis, patients with COVID-19-associated sepsis had lower SIC scores (3.00 [2.00−4.00] vs. 4.00 [3.00−4.00], *p* < 0.001) and SIC positivity rates (11.6% vs. 19.2%, *p* < 0.001) (Table S1).

Among patients with COVID-19-associated sepsis, those with SIC had significantly higher lactate (2.15 mmol/L [1.33−4.60] vs. 1.60 mmol/L [1.20−2.40], *p* < 0.001), PT/INR (1.55 [1.41−2.11] vs. 1.16 [1.08–1.30], *p* < 0.001), PCT (1.45 ng/ml [0.93−21.97] vs. 0.66 [0.23−2.49], *p* < 0.001), APACHE II (32.50 [30.00−33.75] vs. 29.00 [23.00−34.00], *p* = 0.007), as well as higher SOFA scores (12.50 [10.50−15.00] vs. 10.00 [8.00−13.00], *p* < 0.001) (Table S2).

Furthermore, both SIC positivity (OR: 2.229, 95% CI: 1.123−4.423, *p* = 0.022) and increasing SIC score (OR: 1.362, 95% CI: 1.131−1.641, *p* = 0.001) were significantly associated with in-hospital mortality in univariate logistic regression (Table S3).

## Discussion

In this study, SIC was significantly associated with in-hospital mortality and several indicators of clinical severity among patients with sepsis admitted to the ICU. Patients with SIC experienced a more severe clinical course, including higher mortality and greater vasopressor requirements throughout the ICU stay. Furthermore, SIC remained independently associated with in-hospital mortality after multivariable adjustment, suggesting that SIC may reflect both coagulation abnormalities and overall disease severity in critically ill patients with sepsis. Previous studies have shown that thromboinflammatory interactions between the inflammatory and coagulation pathways contribute to microvascular thrombosis and organ dysfunction in sepsis [9, 10, 14, 15]. In this context, SIC may represent an early clinical manifestation of coagulation pathway involvement during the systemic inflammatory response [4].

In our study, three multivariable logistic regression models were constructed to evaluate the association between SIC and in-hospital mortality across different clinical contexts. In the first model, SIC positivity remained independently associated with mortality after adjustment for relevant clinical variables. In the same model, advanced age and lower albumin levels were also independently associated with mortality in patients with sepsis. Previous studies have similarly reported associations between advanced age, hypoalbuminemia, and adverse outcomes in sepsis [9, 16]. In addition, hypoalbuminemia has been associated with endothelial dysfunction and increased disease severity in sepsis [16].

Lactate was also independently associated with mortality in the multivariable analysis. As is well known, lactate is an important marker associated with tissue hypoperfusion and disease severity in sepsis [17], and its association with mortality in sepsis has been demonstrated previously [2]. In our study, patients with SIC exhibited significantly higher lactate levels and lower albumin levels compared to those without SIC. These findings may reflect greater overall disease severity and impaired tissue perfusion in SIC (+) patients.

In the second model, PLT count and PT/INR levels remained independently associated with mortality, whereas fibrinogen and D-dimer levels did not remain statistically significant after adjustment. These findings are generally consistent with the dynamic nature of sepsis-associated coagulopathy [1, 18]. Previous studies have shown associations between thrombocytopenia, prolonged prothrombin time, and adverse outcomes in sepsis [19, 20], whereas fibrinogen levels may initially increase because of the acute phase response, potentially limiting their independent association with mortality in multivariable analyses [21, 22].

In the third model, the SIC score remained independently associated with mortality alongside APACHE II, suggesting a possible relationship between coagulation abnormalities and overall disease severity in patients with sepsis. Previous studies have also reported that the prognostic performance of the SIC score for mortality may be comparable to that of the SOFA score in certain clinical settings [9].

In addition to the multivariable findings, comparison of predictive performance demonstrated that adding the SIC score to APACHE II resulted in a modest but statistically significant improvement in model discrimination. Although the absolute increase in AUC was small, the combined model also showed improved calibration and explained variance compared with APACHE II alone. These findings suggest that the SIC score may provide limited incremental prognostic information beyond conventional severity assessment. However, given the modest magnitude of improvement and the limited discriminative performance of the SIC score alone, it should not be considered a standalone clinically useful mortality prediction model but rather interpreted as a complementary prognostic marker alongside established severity assessment tools such as APACHE II.

In our study, the prevalence of SIC was 19.2%, which was lower than the rates reported in several previous studies. While the reported prevalence of SIC in sepsis has generally ranged between 50% and 70% [6, 13], the HYPRESS study reported a prevalence of approximately 22.1%, which is more consistent with our findings [17]. Differences in SIC prevalence across studies may be related to variations in patient populations, sepsis severity, ICU settings, and diagnostic criteria used to assess SIC.

Additionally, mortality among SIC (+) patients in our cohort was significantly high at 79.3%. Tanaka et al. showed that in-hospital mortality was higher in patients who developed coagulopathy, reporting a rate of 35.8% [13]. The higher mortality observed in our cohort may be explained, at least in part, by the greater severity of illness in this respiratory ICU population, as reflected by the high vasopressor requirement; notably, 98% of SIC (+) patients required vasopressor support within the first 48 h. In addition, our center is a tertiary referral respiratory ICU that predominantly manages patients with advanced respiratory failure, severe pneumosepsis, and multiple comorbid chronic pulmonary diseases, which may have contributed to the high overall mortality observed in this cohort. Previous studies have also reported that sepsis-related mortality may reach 70–90% in selected critically ill patient populations, particularly among those with multiple organ dysfunction [2]. Therefore, the high mortality observed in our cohort should be interpreted within the context of the specific characteristics of this respiratory ICU population and may not be directly generalizable to all patients with sepsis.

Although patients who developed SIC had a shorter ICU LOS, mortality rates were higher in this group. This finding may be related to earlier mortality among critically ill SIC (+) patients. Furthermore, higher CRP and PCT levels observed in SIC (+) patients were consistent with a higher inflammatory burden [2, 23] and may reflect the close interaction between inflammation and coagulation pathways in sepsis.

Most patients in our cohort were diagnosed with pneumosepsis, and previous studies have reported associations between pneumonia-related coagulopathy and higher mortality [24]. Finally, although the SIC score demonstrated limited discriminative performance for predicting mortality in the ROC analysis, patients with SIC scores ≥ 4 had higher mortality rates in our cohort. Therefore, while the SIC score alone should not be considered a standalone clinically useful mortality prediction model, it may still contribute to overall risk assessment when interpreted alongside established severity scores and the broader clinical context.

In our study, patients who developed SIC more frequently required organ support, particularly vasopressor therapy. Previous studies have suggested that microvascular thrombosis and endothelial dysfunction may contribute to impaired organ perfusion in sepsis [6]. In this context, SIC may reflect the coexistence of coagulation abnormalities and greater organ dysfunction severity in critically ill patients with sepsis.

Assessing organ support requirements as clinical outcomes may provide additional insight into the relationship between SIC and disease severity in sepsis. In particular, the coexistence of impaired organ perfusion and coagulation abnormalities in SIC (+) patients may support the need for further prospective studies to determine whether SIC-guided therapeutic strategies, including anticoagulant-based approaches, may benefit selected patient populations [22].

As is well known, early sepsis management primarily consists of fluid resuscitation and appropriate antimicrobial therapy [25]. Previous studies have investigated the potential effects of anticoagulant agents, including heparin, recombinant thrombomodulin, and activated protein C, on coagulation abnormalities and inflammatory pathways in critically ill patients with sepsis [20, 26, 27]. In addition, Japanese guidelines have included recommendations regarding the use of antithrombin and recombinant thrombomodulin in patients with SIC [4]. However, the role of anticoagulant-based therapies in sepsis-associated coagulopathy remains uncertain, and current international sepsis guidelines mainly recommend low-molecular-weight heparin for thromboprophylaxis rather than targeted treatment of SIC. Further randomized controlled studies are needed to clarify the clinical relevance of these approaches [1].

Recent advances in molecular biomarker research suggest that emerging biomarkers, including circulating microRNAs (miRNAs) and plasma gelsolin (pGSN), may further improve prognostic assessment and individualized risk stratification in sepsis-associated coagulopathy. A recent study has demonstrated associations between altered miRNA expression profiles, pGSN levels, disease severity, organ dysfunction, and mortality in patients with sepsis [28]. In addition, these biomarkers may provide complementary information on immune dysregulation, endothelial injury, and thromboinflammatory pathways. However, their clinical utility in sepsis-associated coagulopathy remains investigational, and prospective multicenter studies are needed before they can be integrated into routine clinical practice.

### Limitations

This study has several important limitations. First, its retrospective, single-center design limits the ability to establish causal relationships and may reduce the generalizability of the findings. In addition, the study population consisted predominantly of critically ill patients treated in a tertiary respiratory ICU, many of whom had advanced respiratory failure and severe pneumosepsis. The high severity of illness in this cohort, together with the substantial requirement for vasopressor support, may have contributed to the elevated mortality rates observed. Consequently, these findings may not be representative of the general ICU population or of patients with less severe presentations of sepsis.

Second, potential selection bias should be considered because patients with active hematological malignancies, chronic anticoagulant use, major bleeding, trauma-related ICU admissions, and COVID-19 were excluded from the study. These exclusions may have influenced the cohort’s clinical characteristics and affected the observed prevalence and prognostic performance of SIC.

Third, coagulation parameters and SIC scores were assessed only at baseline, so persistent SIC, worsening SIC, or longitudinal changes in SIC during ICU follow-up could not be evaluated. Recent studies suggest that serial SIC assessment may provide greater prognostic value than a single baseline measurement. Consequently, the prognostic significance of SIC trajectories could not be assessed in the present study and should be addressed in future prospective investigations.

Furthermore, although associations between SIC and organ support requirements were observed, several potentially important confounding factors could not be evaluated because of the retrospective study design. These included fluid balance, corticosteroid use, anticoagulant therapy, administration of antithrombin or recombinant thrombomodulin, timing of antimicrobial therapy, adequacy of source control, ventilatory management strategies, and treatment response. In addition, the influence of infection source and causative microorganisms on mortality outcomes was not fully analyzed. Consequently, residual confounding cannot be excluded despite multivariable adjustment.

In addition, although the SIC score was independently associated with mortality, its discriminative performance in ROC analysis was limited. Therefore, SIC should not be used as a standalone clinical decision-making tool; it should be considered alongside other disease severity assessments.

Finally, the findings were not externally validated in independent multicenter cohorts. Prospective multicenter studies incorporating serial biomarker assessment and external validation are needed to better clarify the clinical relevance of SIC and its relationship to the progression of organ dysfunction in critically ill patients with sepsis.

## Conclusion

In this study, SIC was significantly associated with in-hospital mortality and several indicators of clinical severity in sepsis patients admitted to the ICU. Higher mortality rates were observed among patients who developed SIC, and both SIC positivity and the SIC score remained independently associated with mortality in multivariable analyses after adjustment for disease severity indicators.

In addition, SIC was associated with greater organ support requirements, particularly vasopressor use and escalation to second-line vasopressor therapy. These findings may reflect the relationship between coagulation abnormalities, impaired organ perfusion, and overall disease severity in critically ill patients with sepsis. Although the SIC score demonstrated limited discriminative performance for mortality in ROC analysis, and therefore should not be considered a standalone mortality prediction tool, SIC scores ≥ 4 were associated with higher mortality rates in our cohort.

Overall, these findings suggest that early identification of coagulation abnormalities may provide complementary prognostic information when interpreted alongside established severity assessment tools rather than as a standalone mortality prediction model.

## Supplementary Information

Supplementary Material 1.

## Data Availability

The datasets used and/or analyzed during the current study are available from the corresponding author on reasonable request.

## Abbreviations

SIC: Sepsis-Induced Coagulopathy
DIC: Disseminated Intravascular Coagulation
IMV: Invasive Mechanical Ventilation
CRRT: Continuous Renal Replacement Therapy
ICU: Intensive Care Unit
ICU LOS: Length of Stay in the Intensive Care Unit
COVID-19: Coronavirus Disease-2019
Hgb: Hemoglobin
WBC: White Blood Cell Count
PLT: Platelet Count
PT/INR: Prothrombin Time / International Normalized Ratio
CRP: C-Reactive Protein
PCT: Procalcitonin
APACHE II: Acute Physiology and Chronic Health Evaluation
SOFA: Sequential Organ Failure Assessment
ISTH: International Society on Thrombosis and Haemostasis
SPSS: Statistical Package for the Social Sciences
ROC: Receiver Operating Characteristics
AUC: Area Under Curve
miRNA: MicroRNA
pGSN: Plasma Gelsolin
