# Supplementary material for: Prognostic impact of sepsis-induced coagulopathy on mortality and clinical outcomes in ICU patients
Source: BMC Anesthesiol. 2026 Jul 18;26:620. doi: 10.1186/s12871-026-04104-3 (PMC13670567; doi:10.1186/s12871-026-04104-3)
Supplement: Supplementary file 1 [file 12871_2026_4104_MOESM1_ESM.docx]

**Table S1.** Comparison of coagulation characteristics and clinical outcomes between COVID-19-associated sepsis and non-COVID sepsis patients

| Variables | COVID (+)  Median (Q1-Q3) or  n (%)  n=414 | COVID (-)  Median (Q1-Q3) or  n (%)  n=1305 | p-value |
| --- | --- | --- | --- |
| Gender  Male  Female | 241 (58.2)  173 (41.8) | 728 (55.8)  577 (44.2) | 0.386 |
| Age | 62.00 (47.00-70.00) | 68.00 (56.25-77.75) | **<0.001** |
| Lactate mmol/L | 1.70 (1.20-2.50) | 1.90 (1.30-3.58) | **<0.001** |
| PLT ×10^9^/L | 238.00 (174.00-316.00) | 248.50 (153.00-328.75) | **0.048** |
| Pro-BNP pg/ml | 2210.00 (586.10-8350.00) | 5494.50 (1451.50-18067.25) | **<0.001** |
| D-dimer mg/L | 1.54 (0.84-4.78) | 2.82 (1.52-6.32) | **<0.001** |
| PT/INR | 1.17 (1.09-1.36) | 1.33 (1.21-1.56) | **<0.001** |
| Fibrinogen mg/dl | 529.00 (413.00-685.00) | 501.78 (368.25-677.25) | **<0.001** |
| CRP mg/dl | 135.26 (48.00-203.00) | 123.50 (50.75-238.77) | 0.844 |
| PCT ng/ml | 0.73 (0.23-2.54) | 1.80 (0.64-17.48) | **<0.001** |
| APACHE II | 29.00 (23.00-34.00) | 28.00 (23.00-34.00) | 0.120 |
| SOFA | 10.00 (8.00-13.00) | 10.00 (7.00-13.00) | **0.019** |
| SOFA (with 4 items) | 10.00 (8.00-12.00) | 10.00 (6.00-13.00) | **<0.001** |
| SIC score | 3.00 (2.00-4.00) | 4.00 (3.00-4.00) | **<0.001** |
| SIC (+) | 48 (11.6) | 251 (19.2) | **<0.001** |
| In-hospital mortality | 246 (59.4) | 838 (64.2) | 0.078 |
| ICU LOS days | 11.67 (6.05-19.83) | 5.88 (2.67-11.79) | **<0.001** |

COVID-19: Coronavirus disease-2019, PLT: Platelet, Pro-BNP: Pro-B-type natriuretic peptide, PT/INR: Prothrombin time/International normalized ratio, CRP: C-reactive protein, PCT: Procalcitonin, APACHE II: Acute physiology and chronic health evaluation, SOFA: Sequential organ failure assessment, SIC: Sepsis-induced coagulopathy, ICU LOS: Length of stay in the intensive care unit.

**Table S2.** Comparison of clinical and coagulation characteristics according to SIC status in patients with COVID-19-associated sepsis

| Variables | SIC (+)  Median (Q1-Q3) or  n (%)  n=48 | SIC (-)  Median (Q1-Q3) or  n (%)  n=366 | p-value |
| --- | --- | --- | --- |
| Gender  Male  Female | 35 (72.9)  13 (27.1) | 206 (56.3)  160 (43.7) | **0.028** |
| Age | 54.50 (50.00-76.50) | 62.00 (47.00-70.00) | 0.484 |
| Lactate mmol/L | 2.15 (1.33-4.60) | 1.60 (1.20-2.40) | **<0.001** |
| PLT ×10^9^/L | 147.00 (89.00-148.75) | 241.00 (186.00-333.00) | **<0.001** |
| Pro-BNP pg/ml | 5157.50 (1097.75-8780.50) | 2172.00 (553.10-8350.00) | **<0.001** |
| D-dimer mg/L | 1.13 (0.61-1.55) | 1.80 (0.87-4.93) | 0.069 |
| PT/INR | 1.55 (1.41-2.11) | 1.16 (1.08-1.30) | **<0.001** |
| Fibrinogen mg/dl | 449.50 (357.00-668.25) | 529.00 (416.00-696.00) | **<0.001** |
| CRP mg/dl | 88.65 (29.65-188.75) | 138.00 (53.00-203.00) | 0.853 |
| PCT ng/ml | 1.45 (0.93-21.97) | 0.66 (0.23-2.49) | **<0.001** |
| APACHE II | 32.50 (30.00-33.75) | 29.00 (23.00-34.00) | **0.007** |
| SOFA | 12.50 (10.50-15.00) | 10.00 (8.00-13.00) | **<0.001** |
| SOFA (with 4 items) | 11.50 (9.25-13.50) | 10.00 (8.00-12.00) | **<0.001** |
| SIC score | 5.00 (5.00-5.00) | 2.00 (2.00-3.00) | **<0.001** |
| In-hospital mortality | 36 (75) | 210 (57.4) | **0.019** |
| ICU LOS days | 10.04 (6.23-20.91) | 13.04 (6.33-21.71) | **<0.001** |

COVID-19: Coronavirus disease-2019, PLT: Platelet, Pro-BNP: Pro-B-type natriuretic peptide, PT/INR: Prothrombin time/International normalized ratio, CRP: C-reactive protein, PCT: Procalcitonin, APACHE II: Acute physiology and chronic health evaluation, SOFA: Sequential organ failure assessment, SIC: Sepsis-induced coagulopathy, ICU LOS: Length of stay in the intensive care unit.

**Table S3.** Univariate logistic regression analysis for predictors of in-hospital mortality in COVID-19-associated sepsis patients

| Variables | OR (95% CI) | B coefficient | p-value |
| --- | --- | --- | --- |
| SIC (+) | 2.229 (1.123-4.423) | 0.801 | **0.022** |
| SIC score | 1.362 (1.131-1.641) | 0.309 | **0.001** |

COVID-19: Coronavirus disease-2019, SIC: Sepsis-induced coagulopathy.
